# Pneumococcal Carriage in Young Children One Year after Introduction of the 13-Valent Conjugate Vaccine in Italy

**DOI:** 10.1371/journal.pone.0076309

**Published:** 2013-10-04

**Authors:** Romina Camilli, Laura Daprai, Francesca Cavrini, Donatella Lombardo, Fabio D’Ambrosio, Maria Del Grosso, Maria Fenicia Vescio, Maria Paola Landini, Maria Grazia Pascucci, Erminio Torresani, Maria Laura Garlaschi, Vittorio Sambri, Annalisa Pantosti

**Affiliations:** 1 Department of Infectious, Parasitic and Immune-Mediated Diseases, Istituto Superiore di Sanità, Rome, Italy; 2 Unit of Microbiology, Fondazione IRCCS Cà Granda Ospedale Maggiore Policlinico, Milan, Italy; 3 Unit of Microbiology, S. Orsola- Malpighi Hospital, University of Bologna, Bologna, Italy; 4 Servizio Sanità pubblica, Direzione Generale Sanità e Politiche Sociali, Regione Emilia-Romagna, Bologna, Italy; Centers for Disease Control & Prevention, United States of America

## Abstract

**Background:**

In mid 2010, the 7-valent pneumococcal conjugate vaccine (PCV7) was replaced by the 13-valent conjugate vaccine (PCV13) for childhood immunization in Italy. Our objective in this study was to obtain a snapshot of pneumococcal carriage frequency, colonizing serotypes, and antibiotic resistance in healthy children in two Italian cities one year after PCV13 was introduced.

**Methods:**

Nasopharyngeal swabs were obtained from 571 children aged 0-5 years from November 2011-April 2012. Pneumococcal isolates were serotyped and tested for antimicrobial susceptibility. Penicillin and/or erythromycin non-susceptible isolates were analyzed by Multi Locus Sequence Typing (MLST).

**Results:**

Among the children examined, 81.2% had received at least one dose of PCV7 or PCV13 and 74.9% had completed the recommended vaccination schedule for their age. Among the latter, 57.3% of children had received PCV7, 27.1% PCV13, and 15.6% a combination of the two vaccines. The overall carriage rate was 32.9%, with children aged 6-35 months the most prone to pneumococcal colonization (6-23 months OR: 3.75; 95% CI: 2.19-6.43 and 24-35 months OR: 3.15, 95%CI: 2.36-4.22). A total of 184 pneumococcal isolates were serotyped and divided into PCV7 (5.4%), PCV13 (18.0%), and non-PCV13 (82.0%) serotypes. Serotypes 6C, 24F, and 19A were the most prevalent (10.3%, 8.6%, and 8.1%, respectively). The proportion of penicillin non-susceptible (MIC >0.6 mg/L) isolates was 30.9%, while 42.3% were erythromycin resistant. Non-PCV13 serotypes accounted for 75.4% and 70.8% of the penicillin and erythromycin non-susceptible isolates, respectively.

**Conclusions:**

Our results revealed low rates of PCV7 and PCV13 serotypes in Italian children, potentially due to the effects of vaccination. As the use of PCV13 continues, its potential impact on vaccine serotypes such as 19A and cross-reactive serotypes such as 6C will be assessed, with this study providing a baseline for further analysis of surveillance isolates.

## Introduction

*Streptococcus pneumoniae* (pneumococcus) is an important human pathogen causing a broad spectrum of infections, ranging from upper and lower respiratory tract infections (otitis, sinusitis, and pneumonia) to invasive pneumococcal diseases (IPD), such as meningitis and sepsis. Children under 5 years of age and elderly people represent high-risk groups for serious pneumococcal infections. It was estimated in 2000 that 14.5 million children aged < 5 years were affected by a severe pneumococcal disease, leading to about 11% of all deaths in this age group worldwide [1].

The ecological niche of *S. pneumoniae* is the nasopharynx of healthy persons, predominantly children, which represent the main reservoir for pneumococcal transmission in the community [2]. The rate of colonization is particularly high in the first years of life, with pneumococci being acquired, carried for a period of time, and then cleared in a highly dynamic process [3]. Nasopharyngeal colonization is a prerequisite for developing pneumococcal disease. In a minority of colonized persons, bacteria move from the nasopharynx to the sinuses and middle ear cavity or to the lungs causing respiratory tract infections, or invade the bloodstream causing systemic infections [4]. Infection occurs based on the bacterial virulence factors as well as the chronic or transient immune deficiency status of the host [2]. The antiphagocytic polysaccharide capsule is a major virulence factor and is the target of current pneumococcal vaccines. A limited number of prevalent IPD-causing serotypes, among the more than 90 known serotypes, are included in pneumococcal vaccines. In 2000, a pediatric conjugate vaccine (PCV7) containing the 7 most prevalent serotypes (4, 6B, 9V, 14, 18C, 19F, and 23F) causing diseases in children in North America was licensed. The widespread use of PCV7 led to an overall decrease in IPD due to vaccine serotypes in vaccinated children as well as non-vaccinated persons of all ages [5,6]. Conversely, an increase in the prevalence of non-PCV7 serotypes was documented in most countries where the vaccine was used, both in IPD [7,8] and carriage [9-12]. Higher-valency conjugate vaccines, PCV10 and PCV13, were subsequently formulated to extend the serotype coverage. PCV10 included the PCV7 serotypes and the additional serotypes 1, 5, and 7F; while, PCV13 included the additional serotypes 1, 3, 5, 6A, 7F, and 19A.

In Italy, PCV7 was licensed in 2001 but its use was gradually implemented in Italian regions due to different local immunization strategies [13]. In 2008, PCV7 coverage was 55% nationally [14]. At the beginning of 2010, 14 of the 21 regions actively offered the vaccine to all infants [13], according to the 2 + 1-dose vaccination schedule used in Italy [15]. PCV13 replaced PCV7 in all Italian regions between mid 2010 and 2011 [13]. PCV13 was administered, according to regional immunization programs, to newborns and children who started, but did not complete the 3 doses of PCV7; one PCV13 dose was also recommended for children under 24 months of age who had already received the full PCV7 vaccination series [16].

A thorough evaluation of the impact of PCV7 on the incidence of IPD in Italy was not possible since the nationwide surveillance program for invasive bacterial diseases began in 2007 (http://www.simi.iss.it/meningite_batterica.htm).

However, during the implementation of PCV7, a change in the relative rates of the most common serotypes was observed, due to a reduction of PCV7 serotypes and a concurrent increase in non-PCV7 serotypes, most of which were included in PCV13, such as 1, 7F, and 19A (http://www.simi.iss.it/files/Report_MBI.pdf. Accessed 2013 May 31).

Since nasopharyngeal colonization is necessary for invasive disease, it is critical to understand the effects of vaccines on pneumococcal ecology. Nasopharyngeal isolates reflect strains currently circulating in the community; therefore, changes in the distribution of colonizing serotypes following immunization could predict the prevalent serotypes causing IPD. Thus, surveillance of colonization has become an important component of the vaccination monitoring process. Recently, Simell et al [2] suggested using pneumococcal carriage information in vaccine trials as a marker of vaccine-induced protection, especially in children.

Few studies regarding pneumococcal carriage have been conducted in Italy, and most were performed prior to the introduction of PCV7 [17,18]. These studies revealed a low carriage rate, ranging from 8.6% to 14.9%, with PCV7 serotypes representing 56% to 63% of the isolates [17,18]. A recent study, conducted using molecular methods in children from Genoa in the Liguria region where PCV7 coverage was > 90%, revealed a carriage rate of 50%, with the large majority of isolates being non-PCV7 serotypes [19].

In the present study, we examined the nasopharyngeal carriage in children aged 0-5 years in two large Italian cities, Bologna and Milan, at the beginning of PCV13 use. These two cities are located in two regions of North Italy (Emilia-Romagna and Lombardy, respectively), each with a different policy regarding PCV7 implementation [13]. The data obtained in this carriage study represent a picture of the serotypes circulating in Italy one year after the introduction of PCV13 and provide a baseline for future evaluations of the impact of PCV13.

## Materials and Methods

### Study design

To evaluate pneumococcal carriage in Italian children, a cross-sectional study was conducted between November 2011 and April 2012 in children aged 0-5 years in two Italian cities, Bologna (Emilia-Romagna region) and Milan (Lombardy region), located in regions with different pneumococcal vaccination policies. The Emilia-Romagna region actively provided pneumococcal immunization to all infants since 2006; while, the Lombardy region initially offered vaccination only to disease-specific risk groups of children. Since 2009, Lombardy has offered vaccination to all infants upon the parents’ or pediatricians’ request [13]. As expected, different vaccine coverage rates were observed in the two regions in 2008, with 95% PCV7 coverage in Emilia-Romagna and 27% in Lombardy [14]. Vaccination coverage in Lombardy increased substantially starting in 2009 and reached almost 80% at the time of this study (A. Piatti, personal communication). Children attending routine visits in the first years of life were recruited for the study from the pediatric outpatient clinics of the S. Orsola-Malpighi University Hospital in Bologna and a pediatric practice associated with Hospital Maggiore Policlinico in Milan. Children suffering from respiratory tract infections, other respiratory problems, or chronic illnesses were excluded from the study.

A structured questionnaire was filled in by the pediatrician and was used to collect the children’s demographic data (age, gender, number of family members, presence of young siblings under 6 years, daycare attendance, and presence of at least one smoking parent) and their PCV7/PCV13 vaccination status. Children who had received all the recommended doses for their age, according to the Italian schedule (2+1 doses at 3, 5, and 11/13 months), or who had received a catch-up dose at >1 year of age, were considered completely vaccinated. Children who had not completed the recommended vaccination schedule were classified as partially vaccinated.

### Ethics statement

The study was approved by the Ethics Committee of S. Orsola-Malpighi University Hospital in Bologna (reference number: 1946/2011) and the Ethics Committee of Hospital Maggiore Policlinico in Milan, according to the Italian legislation. Parents or guardians of the participating children provided written informed consent. The procedures followed were in accordance with the European Statements for Good Clinical Practice and the Declaration of Helsinki of the World Medical Association.

### Specimen collection

Nasopharyngeal specimens were obtained using a nylon flocked swab (eSwab, Copan, Brescia, Italy), according to the WHO pneumococcal colonization detection protocol [20]. Swabs were streaked onto 5% sheep blood Columbia agar plates supplemented with 5 µg/mL gentamicin within 18 hours of collection and were incubated overnight at 35°C in 5% CO_2_-enriched air. Swabs were then stored at -80°C in milk-tryptone-glucose-glycerol (STGG) medium (www.cdc.gov/ncidod/biotech/files/pcr-pneumo-carriage-March2010.pdf).

### Bacterial identification and serotyping

Pneumococcal isolates were detected by colony morphology and identification was confirmed by optochin susceptibility and bile solubility testing. Individual isolates were serotyped by latex agglutination and the Quellung reaction using commercially available antisera (Statens Serum Institut, Copenhagen, Denmark).

### Antimicrobial susceptibility testing and genotyping

Susceptibilities to penicillin, ceftriaxone, erythromycin, clindamycin, tetracycline, and chloramphenicol were determined by the antimicrobial gradient strip diffusion method (Etest, bioMérieux, Durham, USA). The breakpoints proposed by the EUCAST (http://www.eucast.org/clinical_breakpoints/. Accessed 2013 May 31) were used. Briefly, for penicillin the meningitis criteria were: susceptible, MIC ≤ 0.06 mg/L; resistant, MIC > 0.06 mg/L. The non-meningitis criteria were: susceptible, MIC ≤ 0.06 mg/L; resistant, MIC > 2 mg/L. For the purpose of this study, all *S. pneumoniae* isolates with a penicillin MIC > 0.06 mg/L were defined as non-susceptible. For ceftriaxone, the breakpoints used were: susceptible, MIC ≤ 0.5 mg/L; resistant, MIC > 2 mg/L. Isolates that were not susceptible to antimicrobials were further analyzed by Multi Locus Sequence Typing (MLST) following the procedures described at the MLST website (http://spneumoniae.mlst.net. Accessed 2013 Jun 20). For each serotype, complete allelic profiles were determined for representative isolates from each of the antibiotic susceptibility groups. Partial MLST profiles were determined for the remaining isolates using a previously described strategy [21]. If the *xpt* and *ddl* alleles corresponded to those obtained for the representative isolate, the same Sequence Type (ST) was assigned. If the *xpt* and *ddl* alleles were divergent, a complete allelic profile was obtained. The software eBURST, available from the MLST website, was used to compare the STs obtained with those available at the MLST website and to define clonal complexes (CCs). Pneumococcal Molecular Epidemiology Network clones (PMEN website: http://www.sph.emory.edu/PMEN/. Accessed 2013 Jun 20) were also identified.

### Statistical analysis

Data were checked for outliers, duplicate records, and distribution of the variables. Age, computed by date of examination minus date of birth, was analyzed as a categorical (0-5; 6-23; 24-35; and 36-71 months) variable. For each variable, missing values were tabulated by age, sex, categories of outcome (colonization status), exposure (vaccination status), and recruitment center. We examined the association between colonization status and vaccination exposure (unvaccinated was the reference category). Potential risk factors for nasopharyngeal carriage of *S. pneumoniae* were identified by univariate analysis. Odds ratios (ORs) and 95% confidence intervals (CIs) for colonization were computed using logistic random effect regression models, with the recruitment center as the random effect. Multivariate analysis was adjusted for other covariates like age, day-care centers attendance, number of siblings younger than 6 years, and smoking status of parents. The effect of potential confounding factors was estimated by fitting a model with and without the variable of interest and by comparing the adjusted and crude estimates. Records with missing values for a variable were excluded for each analysis involving that variable. Statistical analysis was carried out in STATA 10 software (StataCorp).

## Results

### Study population and vaccination status

A total of 571 children, 270 from Bologna and 301 from Milan, aged 0-5 years, were enrolled in the study. The mean age was 35 months (SD 17.15) and 316 (55.3%) participants were male. Overall, 78.9% (451/571) of children attended day care centers, and this percentage was higher (97%) in the 24-71 month old group. Only 30.8% (176/571) of children had siblings aged <6 years and 37.1% (212/571) had at least one smoking parent. The only difference observed between the children populations of the two cities with regard to demographic characteristics was a higher presence of young siblings in Milan, which was borderline significant (p= 0.051).

The vaccination status was known for 531 children: 81.2% (431/531) had received at least one dose of PCV7 or PCV13 and 74.9% (398/531) had completed the recommended vaccination schedule for their age. Specifically, 57.3% (228/398) received PCV7, 27.1% (108/398) PCV13, and 15.6% (62/398) a combination of the two vaccines. According to the Italian schedule [16], children vaccinated with a combination of vaccines receive 2 or 3 doses of PCV7 followed by 1 dose of PCV13 (48/62 children, 77.4%) or 1 or 2 doses of PCV7 followed by 2 doses of PCV13 (14/62 children, 22.6%). By age-adjusted analysis, a statistically significant difference was found in the proportion of vaccinated children between Bologna and Milan. In Bologna, 98.8% of children had received at least one vaccine dose and 97.8% were completely vaccinated compared with 68.1% and 57.5%, respectively, in Milan (p < 0.001).

### *S. pneumoniae* carriage

The overall pneumococcal carriage rate was 32.9% (188/571) and there was no significant difference between Bologna and Milan (34.4% and 31.5%, respectively, p= 0.5). The colonization rate increased from 7.1% in the younger age group (0-5 months) to 33.3% (6-23 months), 33.0% (24-35 months), and 35.1% (36-71 months) in the older aged groups (Table 1). Among colonized children, 71.8% (135/188) were completely vaccinated with PCV7 or PCV13 or a combination of the two, while 5.3% (10/188) were partially vaccinated. Among the non-colonized children, 68.6% (263/383) were completely vaccinated and 6.0% (23/383) were partially vaccinated.

**Table 1:** Univariate and multivariate analysis of risk factors for pneumococcal carriage.

	Total children (N=571)	*S. pneumoniae* carriage	Univariate			Multivariate		
	N (%)	N (%)	OR	(95%CI)	P	OR	(95%CI)	P
*Age-group*								
0 to 5 months	28 (4.90)	2 (7.14)	1			1		
6 to 23 months	132 (23.11)	44 (33.33)	6.50	(5.44; 7.77)	<0.001	3.75	(2.19; 6.43)	<0.001
24 to 35 months	124 (21.71)	41 (33.06)	6.42	(4.70; 8.77)	<0.001	3.15	(2.36; 4.22)	<0.001
36 to 71 months	287 (50.26)	101 (35.19)	7.06	(5.85; 8.52)	<0.001	3.03	(1.00; 9.21)	0.051
*Day-care centres*								
no	120 (21.01)	19 (15.83)	1			1		
yes	451 (78.98)	169 (37.47)	3.55	(2.77; 4.56)	<0.001	2.31	(0.89; 5.98)	0.084
*Young siblings^a^*								
0	392 (68.65)	122 (31.12)	1			1		
≥1	176 (30.82)	65 (36.93)	1.25	(1.21; 1.29)	<0.001	1.20	(0.91; 1.57)	0.193
*Smoking^a^*								
no	356 (62.34)	127 (35.67)	1			1		
yes	212 (37.12)	60 (28.30)	0.71	(0.58; 0.87)	0.01	0.70	(0.61; 0.80)	<0.001
*Vaccination status^b^*								
unvaccinated	100 (17.51)	29 (29.0)	1			1		
PCV7	228 (39.93)	85 (45.21)	1.45	(1.42; 1.49)	<0.001	1.34	(0.97; 1.87)	0.073
PCV7/13 + PCV13	170 (29.77)	50 (37.28)	1.02	(0.87; 1.19)	0.250	1.04	(0.74; 1.43)	0.839
partially vaccinated	33 (5.77)	10 (30.30)	1.06	(0.92; 1.22)	0.870	0.86	(0.72; 1.03)	0.121

^a^ Data for 3 children are missing; ^b^ data for 40 children are missing

In univariate analysis, colonization was associated with age, day-care center attendance, presence of young siblings in the family, and prior vaccination with PCV7 (Table 1). Unexpectedly, the presence of at least one smoking parent was significantly associated with a lower carriage rate, both in univariate and multivariate analyses (Table 1). Further analysis was performed only on children from Milan, for whom information about which parent was the smoker in the household (if mother, father, or both) was available, and indicated that a smoking mother was associated with a higher risk of pneumococcal carriage in children (OR: 3.66; 95%CI: 1.53-8.73, p= 0.003). The multivariate logistic regression model showed children 6-35 months of age were the most prone to pneumococcal colonization (6-23 months, OR: 3.75, 95%CI: 2.19-6.43, p<0.001; 24-35 months, OR: 3.15, 95%CI: 2.36-4.22, p<0.001) (Table 1).

### Serotype distribution

**Figure 1:**
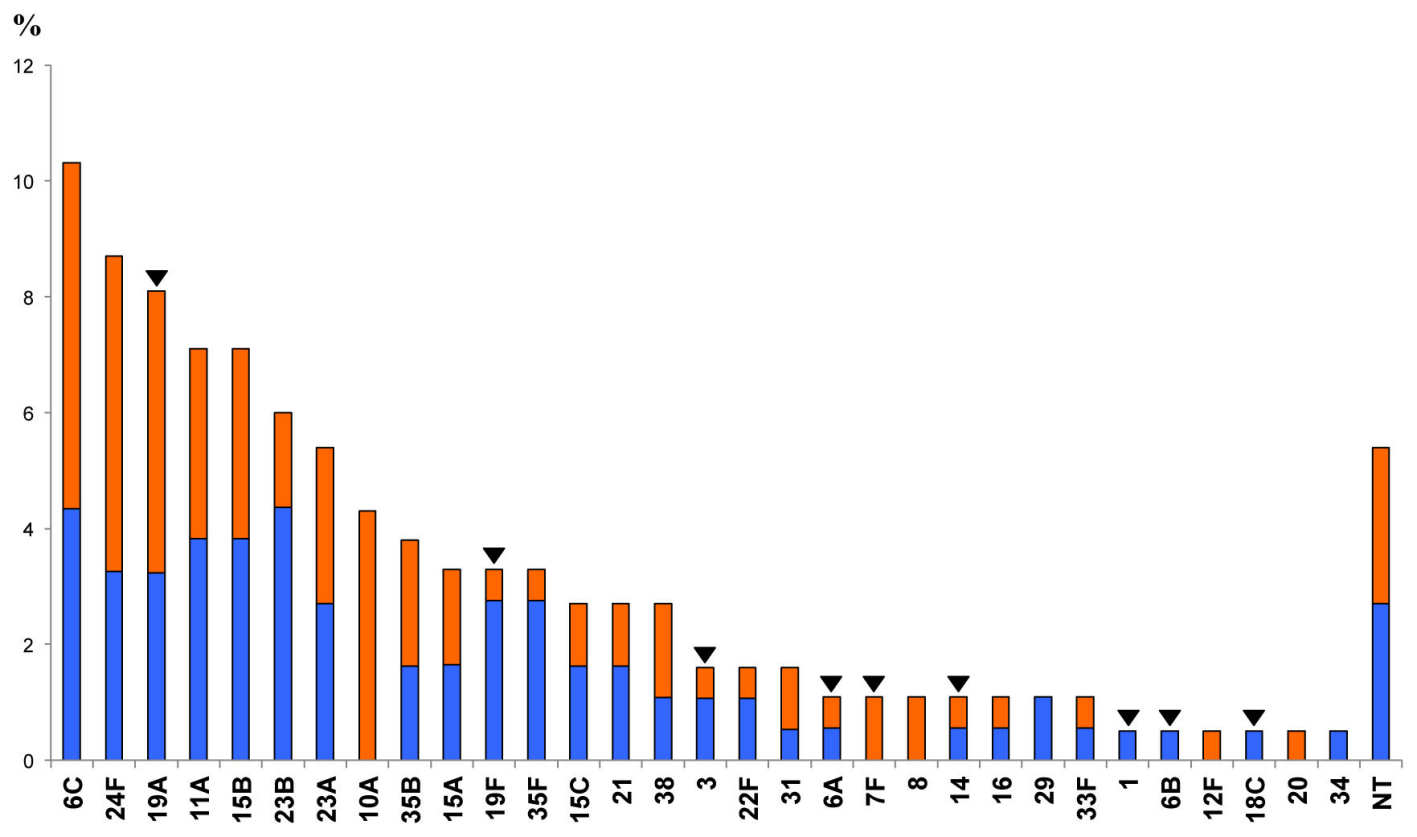
Serotype distribution (in ranking order) of colonizing pneumococci collected from children.

Overall, 189 pneumococcal isolates were obtained from 188 children, as 2 different isolates were obtained in one child. Five isolates were not viable upon storage and could not be serotyped. Of the 184 isolates, 31 different serotypes were identified, with 6C (19/184, 10.3%), 24F (16/184, 8.6%), and 19A (15/184, 8.1%) being the most common; in addition, 5.4% of the isolates (10/184) were non-typeable (NT) (Figure 1). Overall, 5.4% of the isolates were PCV7 serotypes, 18.0% were PCV13 serotypes, and 82.0% non-PCV13 serotypes (Figure 1). No significant difference was observed in the percentage of non-PCV13 serotypes between the children in Bologna and Milan (p= 0.856). By age-adjusted multivariate analysis, children who had received PCV7 were protected from the included serotypes; while, children who had received PCV13 or a combination of PCV7 and PCV13 were protected from the serotypes included in both vaccines. Vaccination with either PCV7 or PCV13 increased the risk of colonization with non-PCV13 serotypes (Table 2).

Within each serotype, the orange portion indicates isolates from Bologna and the blue portion isolates from Milan. The arrowheads indicate PCV13 serotypes.

**Table 2:** Age-adjusted odds ratio of the association between the vaccination status of children and colonization by vaccine serotypes (PCV7 and PCV13) and non-PCV13 serotypes.

	Total children	PCV7 (N = 10)					PCV13 (N = 33)					non-PCV13 (N = 151)			
	N	N	OR	(95%CI)	P		N	OR	(95%CI)	P		N	OR	(95%CI)	P
*Vaccination status*															
unvaccinated	100	5	1				9	1				18	1		
PCV7	228	1	0.13	(0.02; 0.86)	0.034		5	0.34	(0.06; 2.07)	0.242		43	1.59	(0.96; 2.65)	0.073
PCV7/13+PCV13	170	4	0.30	(0.25; 0.37)	<0.001		16	0.69	(0.66; 0.72)	<0.001		69	1.90	(1.40; 2.57)	<0.001
partially vaccinated	33	0	-				0	-				10	1.99	(1.97; 2.01)	<0.001
unknown	40						3					11			

The ranking order of serotypes was slightly shifted between the two populations (Table 3). In particular, the most prevalent serotypes in Bologna were 6C, 24F, and 19A followed by 10A; while, in Milan, serotypes 23B and 6C were the most prevalent, followed by 11A, 15B, 24F, and 19A. In addition, some serotypes were only recovered in one city, with 7F, 8, 10A, 12F, and 20 only in Bologna and 1, 6B, 18C, 29, and 34 only in Milan (Table 3, Figure 1). With the exception of 19A, which was the third most prevalent serotype, other PCV13 serotypes (1, 3, 6A, 6B, 7F, 14, 18C, and 19F) were poorly represented (0.5% to 3.2%, Table 3).

**Table 3:** Serotype distribution in ranking order.

**Serotype**	**Total**					**Bologna**				**Milan**		
	Ranking order	No. isolates	%	Cumulative %		Ranking order	No. isolates	%		Ranking order	No. isolates	%
6C	1	19	10.3	10.3		1	11	11.8		1	8	8.7
24F	2	16	8.6	19.0		2	10	10.7		3	6	6.5
19A	3	15	8.1	27.1		3	9	9.6		3	6	6.5
11A	4	13	7.0	34.2		5	6	6.4		2	7	7.6
15B	4	13	7.0	41.3		5	6	6.4		2	7	7.6
23B	5	11	5.9	47.2		8	3	3.2		1	8	8.7
23A	6	10	5.4	52.7		6	5	5.3		4	5	5.4
NT	6	10	5.4	58.1		6	5	5.3		4	5	5.4
10A	7	8	4.3	62.5		4	8	8.6		-	0	0
35B	8	7	3.8	66.3		7	4	4.3		5	3	3.2
15A	9	6	3.2	69.5		8	3	3.2		5	3	3.2
19F	9	6	3.2	72.8		10	1	1.0		4	5	5.4
35F	9	6	3.2	76.0		10	1	1.0		4	5	5.4
15C	10	5	2.7	78.8		9	2	2.1		5	3	3.2
21	10	5	2.7	81.5		9	2	2.1		5	3	3.2
38	10	5	2.7	84.2		8	3	3.2		6	2	2.1
3	11	3	1.6	85.8		10	1	1.0		6	2	2.1
22F	11	3	1.6	87.5		10	1	1.0		6	2	2.1
31	11	3	1.6	89.1		9	2	2.1		7	1	1.0
6A	12	2	1.0	90.2		10	1	1.0		7	1	1.0
7F	12	2	1.0	91.3		9	2	2.1		-	0	0
8	12	2	1.0	92.3		9	2	2.1		-	0	0
14	12	2	1.0	93.4		10	1	1.0		7	1	1.0
16	12	2	1.0	94.5		10	1	1.0		7	1	1.0
29	12	2	1.0	95.6		-	0	0		6	2	2.1
33F	12	2	1.0	96.7		10	1	1.0		7	1	1.0
1	13	1	0.5	97.2		-	0	0		7	1	1.0
6B	13	1	0.5	97.8		-	0	0		7	1	1.0
12F	13	1	0.5	98.3		10	1	1.0		-	0	0
18C	13	1	0.5	98.9		-	0	0		7	1	1.0
20	13	1	0.5	99.4		10	1	1.0		-	0	0
34	13	1	0.5	100		-	0	0		7	1	1.0
All		184					93				91	

### Antibiotic susceptibility patterns and clonal analysis of resistant isolates

**Figure 2:**
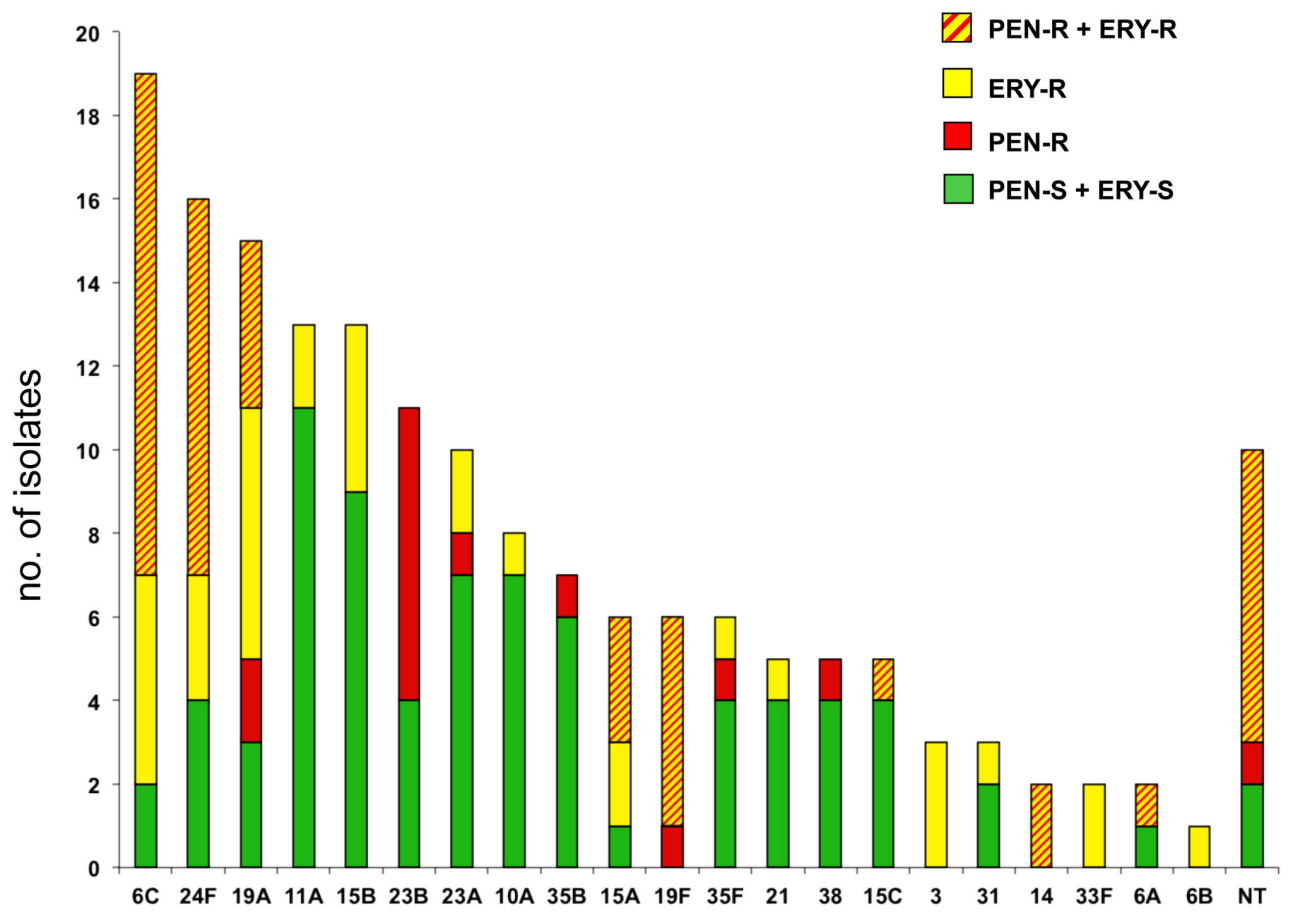
Serotype distribution of penicillin and/or erythromycin non-susceptible isolates.

Using the EUCAST meningitis breakpoints for penicillin (MIC > 0.06 mg/L), 57 isolates out of 184 (30.9%) were penicillin non-susceptible. Of these, 11 (6.0%) showed high penicillin resistance (MIC> 1 mg/L). For the non-meningitis breakpoints, 30.4% (56/184) were intermediate to penicillin (MIC 0.12-2 mg/L), and only 1 isolate (MIC=3 mg/L) was fully resistant (MIC > 2 mg/L). Nine isolates out of 184 (4.8%) were non-susceptible to ceftriaxone (MIC >0.5 mg/L), 8 were intermediate, and only 1 isolate was fully resistant (MIC=3 mg/L). The rates of resistance to the other antibiotics were: erythromycin, 42.3% (78/184); clindamycin, 36.4% (67/184); tetracycline, 36.9% (68/184); and chloramphenicol, 1.6% (3/184). In addition, 23.9% (44/184) were non-susceptible to both penicillin and erythromycin. Non-PCV13 serotypes accounted for 75.4% and 70.8% of the penicillin and erythromycin non-susceptible isolates, respectively. The most prevalent serotypes (6C, 24F, and 19A) included mainly penicillin and/or erythromycin non-susceptible isolates, which accounted for 89.4% of 6C, 75.0% of 24F, and 80.0% of 19A isolates. A high rate of antibiotic non-susceptibility was also present in serotypes 23B, 15A, 19F, 3, 14, 33F, and in NT isolates (Figure 2).

Within each serotype, pneumococcal isolates were divided into 4 categories: susceptible to penicillin and erythromycin (green, PEN-S + ERY-S), non-susceptible to penicillin only (red, PEN-R), non-susceptible to erythromycin only (yellow, ERY-R), and non-susceptible to penicillin and erythromycin (striped, PEN-R + ERY-R).

The 93 isolates that were non-susceptible to penicillin and/or erythromycin were genotyped. A complete MLST profile was obtained for 54 isolates, while a partial MLST profile was used for 39 isolates to infer the ST [21]. In total, 47 STs were detected, clustered in 20 CCs and 2 singleton groups (Table 4). The most abundant CC was CC156, which included 7 STs clustered in different genetic lineages [22] and multiple serotypes (6B, 6C, 14, 19F, 23B, and 24F). The second most abundant CC was CC315, which included PMEN clone Poland^6B^-20/ST315 and comprised only serotype 6C isolates, followed by CC230, which included Denmark^14^-32/ST230 and was composed of serotypes 19A and 24F. The non-susceptible NT isolates belonged to CC344, which included Norway^NT^-42/ST344. Both the fully penicillin resistant isolate (MIC= 3 mg/L) and the ceftriaxone-resistant isolate (MIC= 3 mg/L) were serotype 19A and belonged to ST320/CC320, related to Taiwan^19F^-14/ST236 (Table 4). The other isolates showing high penicillin resistance (MIC> 1 mg/L) belonged mainly to CC230 and CC156. Among the most common serotypes, 6C (5 STs, grouped in 4 different CCs and 1 Singleton group) and 19A (7 STs grouped in 3 different CCs) showed high genetic diversity.

**Table 4:** Genotypic and phenotypic characteristics of the 93 antibiotic-non susceptible *S. pneumoniae* isolates.

CC^a^ (No. isolates)	STs^b^ (No. isolates)	Serotype (No. isolates)	Resistance patterns^c^ (No. isolates)
156 (16)	2372 (4), 7121 (3)	23B (7)	P (7)
	8511 (1)	19F (1)	PT (1)
	162 (4)	24F (4)	PECT (1), ECT (2), EC (1)
	143 (2)	14 (2)	PCxECT (1), PCxEC (1)
	138 (1)	6C (1)	ECT (1)
	469 (1)	6B (1)	ECT (1)
315 (13)	386 (13)	6C (13)	PECT (11), ECT (2)
230 (12)	230 (8)	24F (8)	PECT (8)
	276 (1), 2013 (1), 2674 (1), 9037 (1)	19A (4)	PCxECT (2), PT (2)
344 (8)	344 (5), 3097 (1), 4149 (1), 8448 (1)	NT (8)	PCxECT (1), PECT (5), PET (1), PCx (1)
199 (6)	416 (5), 2343 (1)	19A (6)	ECT (5), EC (1)
63 (6)	63 (1), 374 (1), 2613 (1), 8512 (1), 8872 (1)	15A (5)	PECT (1), PEC (1), PET (1), ECT (1), ET (1)
	2543 (1)	10A (1)	ECT (1)
193 (6)	179 (4)	19F (4)	PECT (4)
	193 (1)	15B (1)	ECT (1)
	1877 (1)	21 (1)	ET (1)
439 (3)	42 (1), 2404 (1), 8449 (1)	23A (3)	ECT (1), EC (1), P (1)
320 (3)	320 (2)	19A (2)	PCxECT (2)^d^
	236 (1)	19F (1)	PCxET (1)
180 (3)	180 (3)	3 (3)	ECTCh (2), ECT (1)
S^e^ (3)	1577 (3)	15B (3)	ECT (3)
62 (2)	62 (2)	11A (2)	ET (1), E (1)
717 (2)	717 (2)	33F (2)	ECT (1), EC (1)
473 (2)	473 (1)	6A (1)	PE (1)
	6759 (1)	6C (1)	ECT (1)
395 (1)	1692 (1)	6C (1)	EC (1)
S^e^ (1)	8447 (1)	6C (1)	PET (1)
393 (1)	3098 (1)	38 (1)	P (1)
460 (1)	446 (1)	35F (1)	P (1)
2991 (1)	2991 (1)	35F (1)	ECT (1)
198 (1)	4346 (1)	35B (1)	P (1)
1262 (1)	8450 (1)	15C (1)	PET (1)
3548 (1)	1766 (1)	31 (1)	E (1)

^a^ Clonal complex (CC) and ^b^ Sequence type (ST) as defined using information from the MLST website (http://spneumoniae.mlst.net); for 54 isolates ST was determined using a complete allelic profile, while for 39 isolates ST was inferred by 2 MLST alleles only (see Materials and Methods); ^c^ For penicillin and ceftriaxone the patterns indicate non-susceptibility (penicillin MIC >0.06 mg/L and ceftriaxone MIC>0.5 mg/L) unless otherwise stated; P: penicillin; Cx: ceftriaxone; E: erythromycin; C: clindamycin; T: tetracycline; Ch: chloramphenicol; ^d^ The pattern indicates one isolate fully resistant to penicillin and one isolate fully resistant to ceftriaxone; ^e^ Singleton.

## Discussion

Surveillance of pneumococcal carriage is a good method for monitoring the impact of pneumococcal conjugate vaccines at the population level [2]. This study evaluated circulating pneumococci among young Italian children at the end of PCV7 use and the beginning of PCV13. Two Italian cities were selected for this study; although, the participants were considered a single homogeneous population since there was no significant demographic difference between the two groups.

In studies conducted in other countries, the pneumococcal carriage rate in young children ranged from 2% to more than 80%, depending on the geographical areas, the socio-economic conditions, and the methods used in the carriage study, including the sampling site (nasopharynx versus oropharynx) [4,9-12,19,23]. Both cultural and molecular methods have been used for carriage studies. Molecular methods, based on pneumococcal DNA detection, are able to identify a higher number of carriers than traditional cultural methods and can allow identification of multiple colonizing serotypes [24,25]. However, cultural methods provide viable bacteria that can be submitted for further characterization, including antimicrobial susceptibility testing.

In our study, the pneumococcal carriage rate was 32.9%, which is consistent with the rates reported in recent studies using cultural methods [12,23]. Our results showed that children aged 6-35 months had the highest potential risk of colonization by pneumococcus. As expected, day-care attendance and the presence of young siblings were associated with an increased prevalence of carriage; although, multivariate analysis showed these were not independent factors. Unexpectedly, we found a decreased risk of carriage in association with the presence of at least one smoking parent. This finding is in contrast to the results of several studies that reported passive smoking represents a risk factor for pneumococcal disease and carriage [26-28]. A more detailed analysis of the children from Milan revealed the risk of pneumococcal colonization increased if the mother was the smoking parent. We do not have an explanation for this association and presume other factors that we have not investigated play a role.

The proportion of children completing the recommended vaccinations for their age with PCV7, PCV13, or a combination of the two was 74.9%. There was a difference in the immunization rates between Bologna and Milan (97.8% versus 57.5%), which likely reflects the different vaccination strategies implemented in the two regions [13]; although, this difference did not appear to influence the pneumococcal colonization rate in this study.

A total of 31 different serotypes were found, with 7 (6C, 24F, 19A, 11A, 15B, 23B, and 23A) representing the majority of those detected; in addition, 5.4% and 17.9% were PCV7 and PCV13 serotypes, respectively. The low rate of PCV13 serotypes was expected since some (1, 3, 5, and 7F) are rarely found in the nasopharynx, irrespective of vaccination, likely because they colonize only for a short period of time and at a low density [2]. Conversely, non-PCV13 serotypes accounted for 82.0% of the isolates, with 6C and 24F being the most abundant.

This study was a cross-sectional analysis of nasopharyngeal carriage in the Italian pediatric population; therefore, we could not evaluate the changes in serotypes distribution following vaccination. However, variations could be inferred from carriage studies performed in Italy before PCV7 implementation, when more than 50% of the serotypes detected in children were PCV7 ones [17,18]. The serotype distribution found in this study is in accordance with several carriage studies performed after PCV7 implementation in different countries, and essentially reflects the effects of PCV7 vaccination [10-12,19,23,29-33]. The additional effect of PCV13 could not be evaluated in this study due to the cross-sectional design and the recent introduction of the vaccine.

PCV7 and PCV13 appeared to be protective against the collective PCV7 and PCV13 serotypes, however protection against individual vaccine serotypes could not be evaluated due to the low number of isolates. In time, the impact of PCV13 on the pneumococcal reservoir will become more evident, and will likely lead to a decline in serotype 19A, which was still prominent in our study. Future studies can also monitor the effect of PCV13 on serotype 6C, which was most common in our study. Cross-reaction between serotype 6A and 6C has been demonstrated in vitro [34] and cross-protection has been shown in a clinical study in France, which reported a decrease in serotype 6C nasopharyngeal carriage in children with acute otitis media who had received at least 1 dose of PCV13 [35].

Serotype 6C was circulating in the pre-vaccine era in many parts of the world [36-38]; although, there was an increase in this serotype after PCV7 implementation, both in carriage [39,40] and IPD [41,42]. A retrospective analysis of isolates from a carriage study performed in Rome in 1999 [18] did not identify any serotype 6C (unpublished data); while, 6C was the most common serotype in our study. Genotyping confirmed there is remarkable genetic heterogeneity of this serotype [36]; although, the majority of the isolates belonged to CC315/ST386, a colonizing clone with multidrug resistance.

The high prevalence of serotype 24F among Italian children is not surprising since this serotype was circulating in our country and a cause of IPD prior to PCV7 [43], and increased significantly after vaccine use [44]. This serotype also increased after PCV7 was introduced in other European countries both in carriage [10,11] and IPD [45]. In our study, serotype 24F was mainly associated with the multidrug resistant CC230, which also included serotype 19A. There was also an increase in the incidence of serotype 19A worldwide, both in IPD [21,46-48] and carriage [9,11], which was at least partly due to serotype replacement after implementation of PCV7. In Italy, serotype 19A increased significantly in IPD mainly due to the expansion of pre-existing clones, such as ST416/CC199, related to Netherlands^15B^-37/ST199 [44]. This same clone was predominant among serotype 19A isolates from our study that were antibiotic resistant. Serotype 19A isolates were also associated with CC320, related to Taiwan^19F^-14/ST236, another multidrug resistant clone [46].

Overall, half of the colonizing pneumococci were antibiotic non-susceptible. With the exception of serotype 19A, most of these isolates had non-PCV13 serotypes, such as 6C, 24F, and 15A. Although only one isolate had a penicillin MIC >2 mg/L and was considered fully resistant to penicillin, the rate of penicillin non-susceptibility was significantly higher than that reported for IPD isolates in Italy (31% versus 14%) [49]. Similarly, the rate of erythromycin resistance was higher than that found in clinical isolates (41% versus 27%) [50]. Since antibiotic use in the period preceding enrollment was not investigated, we could not evaluate if there was a correlation between treatment and an increased prevalence of antimicrobial-resistant pneumococci.

There are some limitations of this study. This was a cross-sectional study of nasopharyngeal carriage; therefore, it is not possible to evaluate changing trends in serotypes or antibiotic resistance, but only to obtain a snapshot of the current situation. In addition, colonization of multiple pneumococcal isolates was not investigated; therefore, some serotypes may have been underestimated. Multiple isolates have been reported in 1.5-50% of carriers [20,23,24,51]; although, a recent study in Portugal demonstrated a decrease in the rate of co-colonization in PCV7-vaccinated children [25]. There was also a limitation in the MLST protocol, as ST was inferred on the basis of only 2 MLST alleles for a portion of the isolates. Due to the recombination ability of *S. pneumoniae*, some isolates had divergent alleles compared with the reference isolate within the same serotype/antibiotic susceptibility group. This could lead to some inaccurate ST allocation, although the CC assignment should not be affected [21].

In conclusion, this study analyzed the pneumococci colonizing healthy children one year after PCV13 was introduced in Italy. It is important to continue to monitor vaccine impact and coverage, using the baseline established here for future comparisons of pneumococcal serotypes.
